# Evaluations of Quinone/Hydroquinone Couples Acting as Two Hydrogen Atoms Antioxidants, Radical Quenchers, and Hydrogen Atom Abstractors

**DOI:** 10.3390/biom15111606

**Published:** 2025-11-15

**Authors:** Xiaotang Chen, Jun-Ke Wang, Xiao-Qing Zhu, Guang-Bin Shen

**Affiliations:** 1 College of Medical Engineering, Jining Medical University, Jining 272067, China; 2 Department of Chemistry, Nankai University, Tianjin 300071, China

**Keywords:** thermodynamic evaluations, quinone/hydroquinone couples, antioxidants, radical quenchers, hydrogen atom abstractors

## Abstract

Quinone/hydroquinone couples play a crucial role in a variety of biochemical processes and chemical syntheses. Extending from our previous work, a practical dataset including the thermodynamic driving forces of 12 chemical processes for 118 quinone/hydroquinone couples accepting or releasing two hydrogen atoms in DMSO is established. The dataset serves as a foundation for assessing and discussing the thermodynamic capabilities of hydroquinones acting as two-hydrogen-atoms antioxidants or radical quenchers, quinones and semiquinone radicals acting as hydrogen atoms abstractors, and quinone/hydroquinone couples acting as dehydrogenation and hydrogenation reagents. The fundamental thermodynamic knowledge is expected to further promote the broader application of quinone/hydroquinone couples in the field of chemical antioxidation and redox reactions.

## 1. Introduction

Quinone/hydroquinone (Q/QH_2_) couples play essential roles in the field of biochemistry [1,2,3,4,5], pharmacochemistry [6] and synthetic chemistry [7,8,9]. Within biological systems, quinone enzymes and their reduced states serve as hydrogen mediators to achieve the delivery of electrons, and hydrogen atoms or ions [1,2,3,4,5]. Propyl gallate, protocatechuic acid (PCA) and nordihydroguaiaretic acid, which feature a distinctive catechol skeleton, are renowned for their antioxidant properties and widely used as food additives to combat oxidation by donating hydrogen atoms to neutralize radicals [10,11,12]. In chemical reactions, Q/QH_2_ couples operate as catalysts [13,14,15,16], particularly in electrochemical syntheses [17,18,19,20,21], where they generally act as hydrogen atoms or hydrides abstractors to initiate substrate activation or complex molecular transformations. Moreover, some well-known quinones [22,23], 2,3-dichloro-5,6-dicyano-*p*-benzoquinone (DDQ) and tetracyano-*p*-benzoquinone, have been applied as dehydrogenation reagents to construct unsaturated bonds by oxidating amines, alcohols, alkanes and pre-aromatic compounds into corresponding imines, aldehydes or ketones, alkenes, and aromatic compounds. Additionally, a variety of synthetic methods are developed or designed to oxidize hydroquinones into corresponding quinones with potential physiological activities [6,24] and reduce quinones into related hydroquinones with potential antioxidant reactivities [10,25]. What is more, Q/QH_2_ couples have already been proved to be a type of potential chemical hydrogen storage materials with the help of the electrochemical method [17,18,19,20,21,26].

It is found that the realization of redox functions for quinones and hydroquinones typically involves H_2_ acceptance and release with Q/QH_2_ couples playing the role of hydrogen mediators. Therefore, the thermodynamics on hydrogenation and dehydrogenation of quinone/hydroquinone couples as hydrogen mediators are critical physical parameters that provide a quantitative assessment of the thermodynamic capabilities of Q/QH_2_ couples acting as redox catalysts or reagents, antioxidants, and potential hydrogen storage materials.

In our previous study, the bond dissociation energies (BDEs) of various hydroquinones (QH_2_) and semiquinone radicals (Q_b_H^•^) in DMSO were calculated by DFT (density functional theory) methods [27], which lays groundwork and presents us a unique opportunity to thoroughly clarify the thermodynamic properties of Q/QH_2_ couples. There are several reasons why DMSO was chosen as the solvent in this study. First, a considerable amount of thermodynamic data for hydroquinones has been reported in DMSO. These extensive experimental datasets allow us to verify the accuracy of our DFT calculations [27]. Second, the Gibbs homolytic dissociation energies of common antioxidants and Y–H bonds are typically determined in DMSO, which facilitates straightforward thermodynamic comparisons. Third, many antioxidant experiments have been evaluated in DMSO. The thermodynamic data provided for quinone/hydroquinone couples are thus useful for selecting appropriate antioxidants. Fourth, DMSO is an excellent reaction solvent. Numerous hydrogen-atom-abstraction-initiated reactions are conducted in DMSO. Therefore, the thermodynamic parameters calculated here can offer practical guidance for using quinone/hydroquinone couples as radical quenchers or hydrogen atom abstractors.

**Scheme 1:**
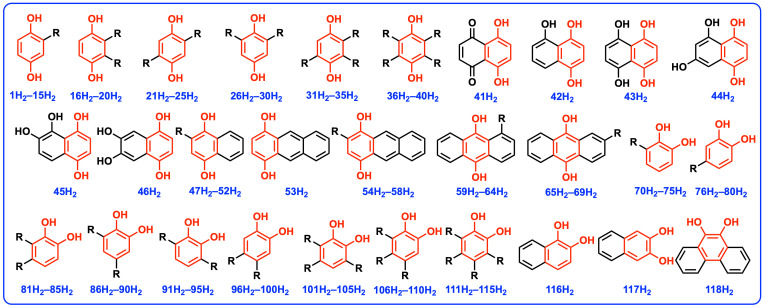
The chemical structures of 118 quinones investigated in this work.

In this study, the thermodynamic properties of 118 hydroquinones are investigated. These hydroquinones encompass 69 *p*-hydroquinones (**1**H_2_–**69**H_2_, denoted as Q*_p_*H_2_) and *o*-hydroquinones (**70**H_2_–**118**H_2_, denoted as Q*_o_*H_2_), and the quinone forms of 118 hydroquinones are depicted in Scheme 1. Thermodynamic driving forces of 12 chemical processes for Q/QH_2_ couples accepting or releasing two hydrogen atoms in DMSO are derived or calculated in accordance with Hess’s law [28]. The resulting thermodynamic data enable us to examine and clarify the thermodynamic properties on hydrogenation and dehydrogenation of quinone/hydroquinone couples as hydrogen mediators. It should be noted that the thermodynamic driving force is a key factor in determining the kinetic process and it can only give thermodynamic guidance and judgment.

## 2. Materials and Methods

### 2.1. Thermodynamic Parameters

**Scheme 2:**
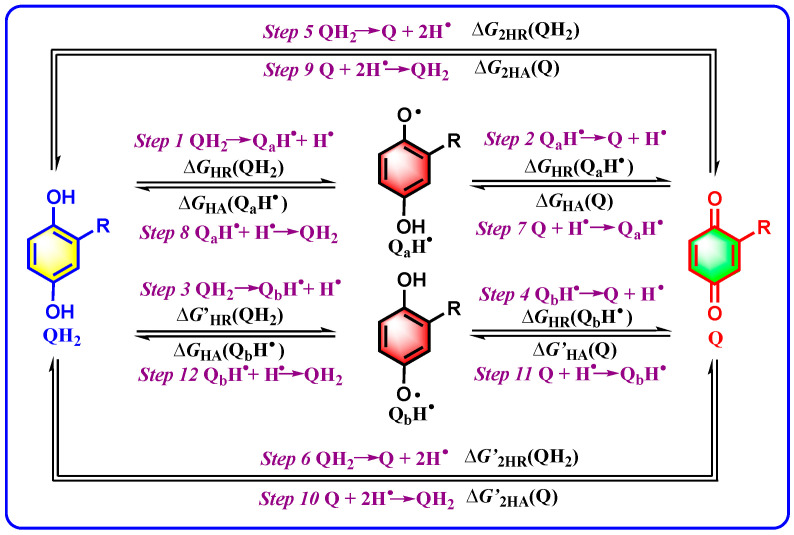
Thermodynamic cycles constructed based on the chemical processes of hydrogenation and dehydrogenation for quinone/hydroquinone couples.

According to the chemical transformations involving the hydrogenation and dehydrogenation of quinone/hydroquinone couples, a thermodynamic analysis platform [28] is established and illustrated in Scheme 2. For a chemical process, the corresponding Gibbs free energy stands out as an essential thermodynamic parameter to judge the spontaneity and quantify the equilibrium constant, as well as assess the stability of initial reactants [28]. In this work, the fourteen Gibbs free energies associated with the hydrogenation and dehydrogenation of Q/QH_2_ couples are derived to provide a detailed quantification of the thermodynamic capabilities and chemical characteristics of Q, QH_2_, and related intermediates, QH^•^.

For the chemical reaction of asymmetric QH_2_ releasing two hydrogen atoms or H_2_, the reaction unfolds in a series of discrete steps (*Steps 1–6*), and the thermodynamic capabilities are quantified by the corresponding Gibbs free energy changes in *Steps 1–6*. *Step 1* is the chemical process of QH_2_ releasing a hydrogen atom to generate semiquinone radical Q_a_H^•^ owning more substituents close to the resulting O-radical center, QH_2_ → Q_a_H^•^ + H^•^, and the thermodynamic capability is described as the corresponding Gibbs free energy, Δ*G*_HR_(QH_2_). *Step 2* is the chemical process of Q_a_H^•^ releasing a hydrogen atom to generate Q, Q_a_H^•^ → Q + H^•^, and the thermodynamic capability is described as the related Gibbs free energy, Δ*G*_HR_(Q_a_H^•^). *Step 3* is the chemical process of QH_2_ releasing a hydrogen atom to generate semiquinone radical Q_b_H^•^ owning less substituents close to the resulting O-radical center, QH_2_ → Q_b_H^•^ + H^•^, and the thermodynamic capability is measured by the corresponding Gibbs free energy, Δ*G′*_HR_(QH_2_). *Step 4* is the chemical process of Q_b_H^•^ releasing a hydrogen atom to generate Q, Q_b_H^•^ → Q + H^•^, and the thermodynamic capability is measured by the related Gibbs free energy, Δ*G*_HR_(Q_b_H^•^). *Step 5* is the chemical process of QH_2_ successively releasing two hydrogen atoms via *Step 1* and *Step 2* to produce Q, QH_2_ → Q + 2H^•^, and the thermodynamic capability is measured by the related Gibbs free energy, Δ*G*_2HR_(QH_2_). *Step 6* is the chemical process of QH_2_ successively releasing two hydrogen atoms via *Step 3* and *Step 4* to produce Q, QH_2_ → Q + 2H^•^, and the thermodynamic capability is measured by the related Gibbs free energy, Δ*G′*_2HR_(QH_2_).

For the chemical reaction of asymmetric Q accepting two hydrogen atoms or H_2_ to give QH_2_, the reaction unfolds in seven discrete steps too (*Steps 7–12*), and the thermodynamic capabilities are quantified by the corresponding Gibbs free energy changes in *Steps 7–12*. *Step 8* is the chemical process of Q accepting a hydrogen atom to generate Q_a_H^•^ owning more substituents close to the resulting O-radical center, Q + H^•^ → Q_a_H^•^, and the thermodynamic capability is described as the corresponding Gibbs free energy, Δ*G*_HA_(Q). *Step 7* is the chemical process of Q_a_H^•^ accepting a hydrogen atom to generate QH_2_, Q_a_H^•^ + H^•^ → QH_2_, and the thermodynamic capability is described as the related Gibbs free energy, Δ*G*_HA_(Q_a_H^•^). *Step 10* is the chemical process of Q accepting a hydrogen atom to generate Q_b_H^•^ owning less substituents close to the resulting O-radical center, Q + H^•^ → Q_b_H^•^, and the thermodynamic capability is measured by the corresponding Gibbs free energy, Δ*G′*_HA_(Q). *Step 9* is the chemical process of Q_b_H^•^ accepting a hydrogen atom to generate QH_2_, Q_b_H^•^ + H^•^ → QH_2_, and the thermodynamic capability is measured by the related Gibbs free energy, Δ*G*_HA_(Q_b_H^•^). *Step 11* is the chemical process of Q successively accepting two hydrogen atoms by *Step 8* and *Step 7* to produce QH_2_, Q + 2H^•^ → QH_2_, and the thermodynamic capability is measured by the related Gibbs free energy, Δ*G*_2HA_(Q). *Step 12* is the chemical process of Q successively accepting two hydrogen atoms by *Step 11* and *Step 10* to produce QH_2_, Q + 2H^•^ → QH_2_, and the thermodynamic capability is measured by the related Gibbs free energy, Δ*G′*_2HA_(Q).

All the definitions on thermodynamic driving forces of 12 chemical processes for Q/QH_2_ couples accepting or releasing two hydrogen atoms in DMSO are collected and shown in Table 1.

### 2.2. Calculation and Acquisition of Thermodynamic Data

Δ*G*_HR_(QH_2_) for *Step 1* can be derived from the enthalpy change of QH_2_ releasing a hydrogen atom to produce Q_a_H^•^, Δ*H*_HR_(QH_2_). The entropy change (*T*Δ*S*) of QH_2_ or QH^•^ releasing a hydrogen atom was estimated as 4.9 kcal/mol at 298 K in DMSO [29]; therefore, the Δ*G*_HR_(QH_2_) values in DMSO could be estimated from *Equation (1)*, Δ*G*_HR_(QH_2_) = −Δ*G*_HA_(Q_a_H^•^) = Δ*H*_HR_(QH_2_) − 4.9 kcal/mol [29]. The thermodynamic relation between Gibbs free energy and enthalpy change for hydrogen atoms transfer is verified many times in our previous work [30]. In addition, due to the fact that the chemical processes of QH_2_ releasing a hydrogen atom to produce Q_a_H^•^ and Q_a_H^•^ accepting a hydrogen atom (*Step 1*: QH_2_ → Q_a_H^•^ + H^•^ and *Step 7*: Q_a_H^•^ + H^•^ → QH_2_) are opposite, the Δ*G*_HA_(Q_a_H^•^) values for *Step 7* could also be obtained by *Equation (1)*, −Δ*G*_HA_(Q_a_H^•^) = Δ*G*_HR_(QH_2_) = Δ*H*_HR_(QH_2_) − 4.9 kcal/mol. Similarly, the values of Δ*G*_HR_(Q_a_H^•^) for *Step 2* and Δ*G*_HA_(Q) for *Step 8* can be derived from *Equation (2)*, Δ*G*_HR_(Q_a_H^•^) = −Δ*G*_HA_(Q) = Δ*H*_HR_(Q_a_H^•^) − 4.9 kcal/mol. The values of Δ*G′*_HR_(QH_2_) for *Step 3* and Δ*G*_HA_(Q_b_H^•^) for *Step 9* can be derived from *Equation (3)*, Δ*G′*_HR_(QH_2_) = −Δ*G*_HA_(Q_b_H^•^) = Δ*H′*_HR_(QH_2_) − 4.9 kcal/mol. The values of Δ*G*_HR_(Q_b_H^•^) for *Step 4* and Δ*G′*_HA_(Q) for *Step 10* can be derived from *Equation (4)*, Δ*G*_HR_(Q_b_H^•^) = −Δ*G′*_HA_(Q) = Δ*H*_HR_(Q_b_H^•^) − 4.9 kcal/mol. The details of DFT calculations of Δ*H*_HR_(QH_2_), Δ*H*_HR_(Q_a_H^•^), Δ*H′*_HR_(QH_2_) and Δ*H*_HR_(Q_b_H^•^) were available in our previous work [27].

Δ*G*_2HR_(QH_2_) for *Step 5* can be obtained from *Equation (5)*, Δ*G*_2HR_(QH_2_) = Δ*G*_HR_(QH_2_) + Δ*G*_HR_(Q_a_H^•^), by constructing a thermodynamic cycle (*Step 1*–*Step 2*–*Step 5*) based on Hess’s law [28]. Similarly, Δ*G*_2HA_(Q) for *Step 11* can also be obtained from *Equation (5)*, Δ*G*_2HR_(QH_2_) = −Δ*G*_2HA_(Q) = Δ*G*_HR_(QH_2_) + Δ*G*_HR_(Q_a_H^•^).

Δ*G′*_2HR_(QH_2_) for *Step 6* can be obtained from *Equation (6)*, Δ*G′*_2HR_(QH_2_) = Δ*G′*_HR_(QH_2_) + Δ*G*_HR_(Q_b_H^•^), by constructing a thermodynamic cycle (*Step 3*−*Step 4*−*Step 6*) based on Hess’s law [28]. Similarly, Δ*G*_2HA_(Q) for *Step 13* can also be obtained from *Equation (6)*, Δ*G′*_2HR_(QH_2_) = −Δ*G′*_2HA_(Q) = Δ*G′*_HR_(QH_2_) + Δ*G*_HR_(Q_b_H^•^).

All the expressions of *Equations (1)*−*(6)* and data sources on fourteen thermodynamic driving forces of Q/QH_2_ couples accepting or releasing two hydrogen atoms in DMSO are illustrated in Table 2.

## 3. Results

In our previous work [27], the values of Δ*H*_HR_(QH_2_) for *Step 1*, Δ*H*_HR_(Q_a_H^•^) for *Step 2*, Δ*H′*_HR_(QH_2_) for *Step 3*, and Δ*H*_HR_(Q_b_H^•^) for *Step 4* of 118 important QH_2_ and their intermediates Q_a_H^•^ and Q_b_H^•^ releasing a hydrogen atom in DMSO were calculated using DFT method. These original values of Δ*H*_HR_(QH_2_), Δ*H*_HR_(Q_a_H^•^), Δ*H′*_HR_(QH_2_), and Δ*H*_HR_(Q_b_H^•^) are displayed in Table S1 of Supplementary Materials.

Building upon this foundation, in this work, the values of Δ*G*_HR_(QH_2_) for *Step 1*, Δ*G*_HR_(Q_a_H^•^) for *Step 2*, Δ*G′*_HR_(QH_2_) for *Step 3*, and Δ*G*_HR_(Q_b_H^•^) for *Step 4* of 118 important QH_2_ and their intermediates, Q_a_H^•^ and Q_b_H^•^, releasing a hydrogen atom, as well as the values of Δ*G*_HA_(Q) for *Step 7*, Δ*G*_HA_(Q_a_H^•^) values for *Step 8*, Δ*G′*_HA_(Q) for *Step 11*, and Δ*G*_HA_(Q_b_H^•^) for *Step 12* of 118 Q and their intermediates Q_a_H^•^ and Q_b_H^•^ accepting a hydrogen atom in DMSO, were estimated by *Equations (1)−(4)*, through considering the entropy change (*T*Δ*S*) of QH_2_ or QH^•^ releasing a hydrogen atom in DMSO (4.9 kcal/mol) [29,30,31].

In this work, Δ*G*_2HR_(QH_2_) for *Step 5*, and Δ*G′*_2HR_(QH_2_) for *Step 6* of 118 QH_2_ releasing two hydrogen atoms, as well as the values of Δ*G*_2HA_(Q) for *Step 9*, and Δ*G*_2HA_(Q) for *Step 10* of 118 Q accepting two hydrogens in DMSO, were derived by *Equations (5) and (6)* based on Hess’s law.

A dataset encompassing all the thermodynamic results of 118 important Q/QH_2_ couples accepting or releasing two hydrogen atoms about 12 chemical steps into DMSO is shown in Table 3.

## 4. Discussion

### 4.1. Further Verification of Data Reliability

The reliability of calculated enthalpy changes in a hydrogen atom release from QH_2_ and QH^•^, including Δ*H*_HR_(QH_2_) for *Step 1*, Δ*H*_HR_(Q_a_H^•^) for *Step 2*, Δ*H′*_HR_(QH_2_) for *Step 3*, and Δ*H*_HR_(Q_b_H^•^) for *Step 4*, was verified in our previous work [27]. For 33 substituted phenols, the theoretically predicted enthalpy changes in O−H homolysis fit well with the experimental data (MD = 0.58 and *r* = 0.98) [27].

In this work, the data accuracy is further verified. For the chemical process of QH_2_ releasing two hydrogen atoms, two distinct pathways are involved. Pathway 1 (*Step* 5) is when QH_2_ successively releases two hydrogen atoms by *Step 1* and *Step 2*; *Step 1* QH_2_ → Q_a_H^•^ + H^•^, and *Step 2* Q_a_H^•^ → Q + H^•^. In contrast, pathway 2 (*Step* 6) is when QH_2_ successively releases two hydrogen atoms by *Step 3* and *Step 4*; *Step 3* QH_2_ → Q_b_H^•^ + H^•^, and *Step 4* Q_b_H^•^ → Q + H^•^. Theoretically, if the calculated thermodynamic data is reliable, the energy changes in pathways 1 (*Step* 5) and 2 (*Step* 6) should ideally be the same based on Hess’s law [28]. The energy difference between Δ*G*_2HR_(QH_2_) for *Step 5* and Δ*G′*_2HR_(QH_2_) for *Step 6* is denoted as ΔΔ*G*_2HR_, ΔΔ*G*_2HR_ = Δ*G′*_2HR_(QH_2_) − Δ*G*_2HR_(QH_2_), which are listed in the first column of Table S2 in Supplementary Materials. As expected, the range of ΔΔ*G*_2HR_ is found to be between −0.1 to 0.1 kcal/mol. By inference, the extremely slight difference in energy strongly suggests that the calculated energies for O–H homolysis are indeed reliable, and so are these derived thermodynamic results of the 12 chemical steps (*Steps 1*–*12*) in this work.

### 4.2. Thermodynamic Capabilities of QH_2_ Acting as Antioxidants by Releasing Two Hydrogen Atoms

Hydroquinones possess a unique antioxidant characteristic [10] that sets them apart from conventional antioxidants, such as butylated hydroxytoluene (BHT) [31] and α-tocopherol (TocOH) [31], as well as nicotinamide coenzymes [NAD(P)H] [28] and so on. Unlike these substances, a single hydroquinone molecule has the capacity to sequentially release two hydrogen atoms, thereby neutralizing two radicals in the process as antioxidants. The same antioxidant characteristic occurs in ascorbic acid (AscH_2_) [31], coenzyme Q [1,2,3,4], and hantzsch ester (HEH_2_) [30], all of which are capable of donating two hydrogen atoms during their antioxidant activity. Given this distinctive feature, the thermodynamic data of QH_2_ releasing two hydrogen atoms, Δ*G*_2HR_(QH_2_), becomes a crucial parameter to evaluate their overall antioxidant properties.

**Scheme 3:**
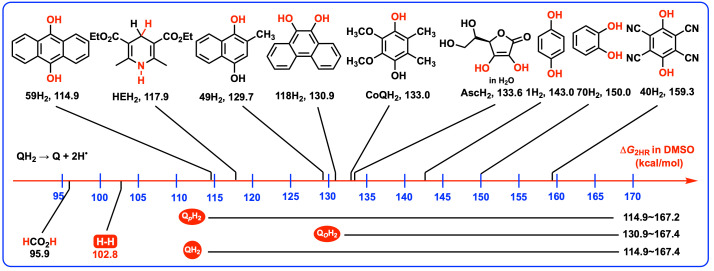
Gibbs free energies of hydroquinones and common reductants successively releasing two hydrogen atoms in DMSO.

Herein, the Gibbs free energies for the sequential release of two hydrogen atoms from hydroquinones and other common reductants in DMSO are presented in Scheme 3 for comparative analysis. A visual examination of Scheme 3 reveals that the Δ*G*_2HR_(QH_2_) scale of 118 QH_2_ spans from 114.9 kcal/mol to 167.4 kcal/mol in DMSO. In particular, for the 69 Q*_p_*H_2_ (**1**H_2_–**69**H_2_), the Δ*G*_2HR_(Q*_p_*H_2_) scale spans from 114.9 kcal/mol to 167.2 kcal/mol, while for the 49 Q*_o_*H_2_ (**70**H_2_–**118**H_2_), the Δ*G*_2HR_(Q*_o_*H_2_) scale spans from 130.9 kcal/mol to 167.4 kcal/mol. It is observed that the Q*_p_*H_2_ (114.9–167.2 kcal/mol) demonstrate a broader thermodynamic window for two-hydrogen-atoms release, compared with Q*_o_*H_2_ (130.9–167.4 kcal/mol). Notably, both Q*_p_*H_2_ (114.9–167.2 kcal/mol) and Q*_o_*H_2_ (130.9–167.4 kcal/mol) share a similar thermodynamic upper limit for two-hydrogen-atoms release in DMSO, capped at approximately 167 kcal/mol. Moreover, 19 Q*_p_*H_2_ (114.9–167.2 kcal/mol), including **43**H_2_ (122.1 kcal/mol), **47**H_2_ (130.7 kcal/mol), **49**H_2_ (129.7 kcal/mol), **53**H_2_–**57**H_2_ (122.2–128.6 kcal/mol), and **59**H_2_–**69**H_2_ (114.9–121.3 kcal/mol) have the better thermodynamic capabilities to release two hydrogen atoms when compared to the entire set of 49 Q*_o_*H_2_ (**70**H_2_–**118**H_2_, 130.9–167.4 kcal/mol) in DMSO. What is more, among these 118 QH_2_, the 9,10-hydroanthraquinones (**59**H_2_–**69**H_2_, 114.9–121.3 kcal/mol) stand out as the most thermodynamically favorable two-hydrogen-atoms antioxidants.

HEH_2_, AscH_2_ and coenzyme Q are recognized for their efficacy as antioxidants capable of donating two hydrogen atoms [30,31], and the Gibbs free energies of HEH_2_ (117.9 kcal/mol) [32,33,34,35,36], AscH_2_ (133.6 kcal/mol in H_2_O) [31] and the close coenzyme Q model 2,3-Me_2_-5,6-MeO_2_-*p*-hydroquinone (CoQH_2_, 133.0 kcal/mol) are displayed in Scheme 3 for comparative purposes. In addition, tetracyano-*p*-benzoquinone (**40**H_2_) is noted for its exceptional oxidizing properties, with a Δ*G*_2HR_(**40**H_2_) value of 159.3 kcal/mol in DMSO. Based on these references, a classification system can be inferred for the thermodynamic strength of QH_2_ acting as two-hydrogen-atoms antioxidants. If a Δ*G*_2HR_(QH_2_) value is less than or equal to 130 kcal/mol, the QH_2_ is categorized as a thermodynamically strong two-hydrogen-atoms antioxidant. If a Δ*G*_2HR_(QH_2_) value is greater than 130 kcal/mol but less than or equal to 150 kcal/mol, the QH_2_ is considered a thermodynamically medium–strong two-hydrogen-atoms antioxidant. If a Δ*G*_2HR_(QH_2_) value exceeds 150 kcal/mol, the QH_2_ is clarified as a thermodynamically weak two-hydrogen-atoms antioxidant. This categorization provides a clear framework for assessing the overall antioxidant potential based on the thermodynamic data of two-hydrogen-atoms donation, offering valuable insights for the design and evaluation of novel antioxidants.

Accordingly, all the 118 QH_2_ (114.9–167.4 kcal/mol) and 69 Q*_p_*H_2_ (114.9–167.2 kcal/mol) cover from the thermodynamically strong, medium–strong, to weak two-hydrogen-atoms antioxidants. The 49 Q*_o_*H_2_ are recognized as the thermodynamically medium-strong or weak two-hydrogen-atoms antioxidants. Upon closer examination, 19 Q*_p_*H_2_ (114.9–167.2 kcal/mol), including **43**H_2_ (122.1 kcal/mol), **47**H_2_ (130.7 kcal/mol), **49**H_2_ (129.7 kcal/mol), **53**H_2_–**57**H_2_ (122.2–128.6 kcal/mol), and **59**H_2_–**69**H_2_ (114.9–121.3 kcal/mol), are recognized as the thermodynamically strong two-hydrogen-atoms antioxidants. The 30 QH_2_ (>150 kcal/mol), including 8 Q*_p_*H_2_ and 22 Q*_o_*H_2_, that is, **12**H_2_ (152.5 kcal/mol), **13**H_2_ (150.2 kcal/mol), **20**H_2_ (152.8 kcal/mol), **25**H_2_ (151.1 kcal/mol), **30**H_2_ (152.7 kcal/mol), **35**H_2_ (155.8 kcal/mol), **40**H_2_ (159.3 kcal/mol), **41**H_2_ (167.2 kcal/mol), **73**H_2_–**75**H_2_ (150.5–151.7 kcal/mol), **79**H_2_ (150.3 kcal/mol), **80**H_2_ (156.9 kcal/mol), **84**H_2_ (150.2 kcal/mol), **85**H_2_ (159.1 kcal/mol), **89**H_2_ (150.6 kcal/mol), **90**H_2_ (158.1 kcal/mol), **93**H_2_–**95**H_2_ (151.1–155.1 kcal/mol), **99**H_2_ (151.3 kcal/mol), **100**H_2_ (162.4 kcal/mol), **103**H_2_–**105**H_2_ (150.7–161.7 kcal/mol), **109**H_2_ (151.4 kcal/mol), **110**H_2_ (164.3 kcal/mol), **114**H_2_ (151.2 kcal/mol), **115**H_2_ (167.4 kcal/mol) and **117**H_2_ (160.2 kcal/mol), are identified as thermodynamically weak two-hydrogen-atoms antioxidants with Δ*G*_2HR_(QH_2_) values exceeding 150 kcal/mol. The remaining 70 QH_2_ fall into the category of thermodynamically medium–strong two-hydrogen-atoms antioxidants. Among all 118 QH_2_, **59**H_2_ (9,10-hydroanthraquinone, 114.9 kcal/mol) is recognized as the most thermodynamically favorable two-hydrogen-atoms antioxidant [10]. In contrast, **115**H_2_, an *o*-hydroquinone with four strong electron-withdrawing cyano groups (167.4 kcal/mol), exhibits the highest Δ*G*_2HR_ value of 167.4 kcal/mol, marking it as the thermodynamically weakest two-hydrogen-atoms antioxidant.

Intriguingly, just like HEH_2_ (117.9 kcal/mol) [35,36], 9,10-hydroanthraquinones (**59**H_2_–**69**H_2_, 114.9–121.3 kcal/mol) exhibit the similar thermodynamic capabilities to release two hydrogen atoms. This similarity in thermodynamic capability to release two hydrogen atoms suggests that 9,10-hydroanthraquinones (**59**H_2_–**69**H_2_) could serve as viable alternatives to HEH_2_ (117.9 kcal/mol) as hydrogen reducers or two-hydrogen-atoms donors in chemical reactions. In particular, **59**H_2_ (114.9 kcal/mol), **61**H_2_ (116.0 kcal/mol), **65**H_2_ (115.0 kcal/mol), and **66**H_2_ (116.0 kcal/mol), are identified as thermodynamically better two-hydrogen-atoms antioxidants or reducers than HEH_2_ (117.9 kcal/mol) in DMSO. These findings underscore and reveal the potential utility of 9,10-hydroanthraquinones as robust antioxidants or reducers in chemical area [10,17,18,19,20,21], particularly in the context of reactions involving DMSO as a solvent.

### 4.3. Thermodynamic Capabilities of QH_2_ and QH^•^ Releasing a Hydrogen Atom as Antioxidants

**Scheme 4:**
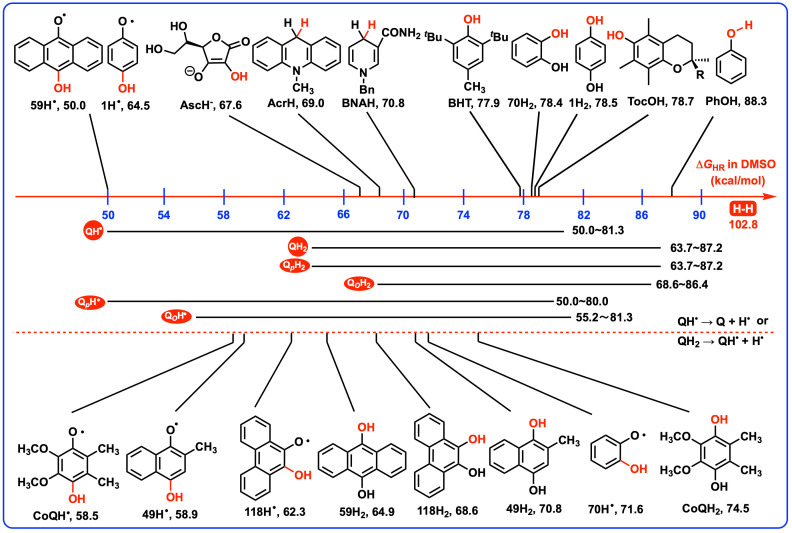
Gibbs free energies of QH_2_, QH^•^, and various X–H bonds releasing a hydrogen atom in DMSO.

As previously discussed, the Δ*G*_2HR_(QH_2_) values reflect the overall antioxidant properties of QH_2_ when releasing two hydrogen atoms. For a given QH_2_, especially for the asymmetric hydroquinones, it is widely known that there exists a huge difference in the thermodynamic capabilities between QH_2_ and QH^•^ releasing a hydrogen atom due to the different levels of stability of QH_2_ and its active intermediate QH^•^. Therefore, the thermodynamic capabilities of QH_2_ and QH^•^ releasing a hydrogen atom, Δ*G*_HR_(QH_2_) and Δ*G*_HR_(QH^•^), are also the crucial thermodynamic parameters for assessing their separate antioxidant potentials of QH_2_ and QH^•^. To delve deeper into these properties, Scheme 4 presents the Gibbs free energies associated with the release of a hydrogen atom from QH_2_, QH^•^, and various O–H bonds in DMSO. By examining these Gibbs free energies, researchers can gain insights into the relative reactivity and stability of QH_2_ and QH^•^, which is essential for understanding their roles in antioxidant processes and for the design of novel antioxidant compounds [10].

As reported in our previous work, [28] a criterion was established for classifying the thermodynamic antioxidant capability. If a Δ*G*_HR_ value is less than or equal to 65 kcal/mol, the QH_2_ or QH^•^ belongs to a thermodynamically strong antioxidant. If a Δ*G*_HR_ value is greater than 65 kcal/mol and less than or equal to 85 kcal/mol, the QH_2_ or QH^•^ belongs to a thermodynamically medium–strong antioxidant. If a Δ*G*_2HR_(QH_2_) value exceeds 85 kcal/mol, the QH_2_ is classified a thermodynamically weak antioxidant.

From Scheme 4, for the first hydrogen atom release, it is clear that the Δ*G*_HR_(QH_2_) values of 118 QH_2_ range from 63.7 to 87.2 kcal/mol, which spans a large scope of 23.5 kcal/mol. To be specific, for *p*-hydroquinones, the Δ*G*_HR_(Q*_p_*H_2_) values of 69 Q*_p_*H_2_ (**1**H_2_–**69**H_2_) also range from 63.7 to 87.2 kcal/mol. Meanwhile, as for *o*-hydroquinones, the Δ*G*_HR_(Q*_o_*H_2_) values of 49 Q*_o_*H_2_ (**70**H_2_–**118**H_2_) range from 68.6 to 86.4 kcal/mol. The Δ*G*_HR_(Q*_p_*H_2_) values (63.7–87.2 kcal/mol) demonstrate a wider thermodynamic spread compared to the Δ*G*_HR_(Q*_o_*H_2_) values (68.6–86.4 kcal/mol). These findings suggest that QH_2_ (63.7–87.2 kcal/mol) are generally considered as the thermodynamically medium–strong antioxidants.

Upon examining the second hydrogen atom release, the Δ*G*_HR_(QH^•^) values of 118 QH^•^ exhibit a substantial range, from 50.0 to 81.3 kcal/mol, which spans an extensive thermodynamic scope of 31.3 kcal/mol. Specifically, for *p*-hydroquinones, the Δ*G*_HR_(Q*_p_*H^•^) values of 69 Q*_p_*H^•^ (**1**H^•^–**69**H^•^) range from 50.0 to 80.0 kcal/mol. In comparison, for *o*-hydroquinones, the Δ*G*_HR_(Q*_o_*H^•^) values of 49 Q*_o_*H^•^ (**70**H^•^–**118**H^•^) range from 55.2 to 81.3 kcal/mol. Similarly, the Δ*G*_HR_(Q*_p_*H^•^) values (50.0–80.0 kcal/mol) demonstrate a wider thermodynamic distribution than Δ*G*_HR_(Q*_o_*H^•^) values (55.2–81.3 kcal/mol). The thermodynamic results suggest that QH^•^ (50.0–81.3 kcal/mol) are clarified as the thermodynamically strong and medium–strong antioxidants. Notably, the thermodynamic antioxidant capabilities of QH^•^ (50.0–81.3 kcal/mol) appear to be enhanced relative to their parent compounds QH_2_, which have Δ*G*_HR_(QH_2_) values ranging from 63.7 to 87.2 kcal/mol.

Further considering the thermodynamic capabilities of 9,10−hydroanthraquinones and their corresponding semiquinone radicals, **59**H_2_–**69**H_2_ and **59**H^•^–**69**H^•^ are generally thermodynamically strong antioxidants with the thermodynamic driving forces of QH_2_ and QH^•^ releasing a hydrogen atom smaller than 65 kcal/mol. Except for the Gibbs free energies of 9,10-hydroanthraquinones (**59**H_2_–**69**H_2_) releasing a hydrogen atom, the Δ*G*_HR_(QH_2_) values of the other 107 QH_2_ exceeds 65 kcal/mol, indicating that these 107 QH_2_ belong to thermodynamically medium–strong and weak antioxidants during the first hydrogen atom release from their hydroquinone states.

For a given QH_2_, if the Δ*G*_HR_(QH_2_) and Δ*G′*_HR_(QH_2_) values are compared with its Δ*G*_HR_(Q_a_H^•^) and Δ*G*_HR_(Q_a_H^•^) values, respectively, the energy differences between them are defined as ΔΔ*G*_HR_ and ΔΔ*G′*_HR_, ΔΔ*G*_HR_ = Δ*G*_HR_(Q_a_H^•^) − Δ*G*_HR_(QH_2_) and ΔΔ*G′*_HR_ = Δ*G*_HR_(Q_b_H^•^) − Δ*G′*_HR_(QH_2_), which are presented in the fourth and fifth columns of Table S2 in Supplementary Materials. The ΔΔ*G*_HR_ and ΔΔ*G″*_HR_ scales could provide insight into the thermodynamic favorability and tendency of each step. [30] It is discovered that the ΔΔ*G*_HR_ and ΔΔ*G″*_HR_ scales range from −26.2 kcal/mol for **26**H_2_ to 2.4 kcal/mol for **117**H_2_, from −24.4 kcal/mol for **26**H_2_ to −3.9 kcal/mol for **76**H_2_ and **102**H_2_ respectively. Except for **42**H_2_ (0.0 kcal/mol) and **117**H_2_ (2.4 kcal/mol), the second hydrogen atom release from QH^•^ is thermodynamically more favorable than the first hydrogen atom release from their parents QH_2_. Furthermore, the majority of ΔΔ*G*_HR_ and ΔΔ*G′*_HR_ values are more negative than −5 kcal/mol, meaning that after QH_2_ releases a hydrogen atom, the following hydrogen atom transfer from generated QH^•^ has greater thermodynamic driving forces.

In addition, for an asymmetric QH_2_, if the Δ*G*_HR_(QH_2_) values for *Step 1* and Δ*G′*_HR_(QH_2_) values for *Step 3* are compared, the energy difference between them is denoted as ΔΔ*G″*_HR_, ΔΔ*G″*_HR_ = Δ*G′*_HR_(QH_2_) − Δ*G*_HR_(QH_2_), which are detailed in the sixth column of Table S2 in Supplementary Materials. The ΔΔ*G″*_HR_ scale ranges from −4.9 kcal/mol for **60**H_2_ to 6.9 kcal/mol for **45**H_2_. This range indicates that the preference for an asymmetric QH_2_ to release a hydrogen atom to form either the semiquinone radical with more substituents adjacent to the nascent O-radical center (Q_a_H^•^) or the one with fewer substituents (Q_b_H^•^) is influenced by the stability differences between Q_a_H^•^ and Q_b_H^•^, which are attributed to the effects of the substituents. This finding highlights the importance of considering the molecular environment surrounding the reactive centers when predicting the antioxidant behavior of hydroquinones and their derived semiquinone radicals.

### 4.4. Thermodynamic Capabilities of QH_2_ and QH^•^ Acting as Radical Quenchers

**Scheme 5:**
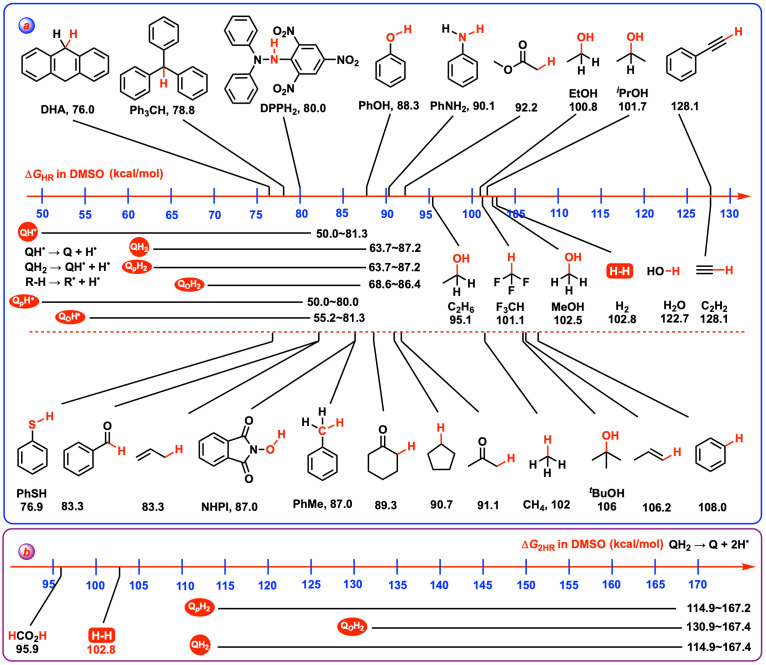
(**a**) Gibbs free energies of hydroquinones and semiquinones releasing hydrogen atoms, along with Gibbs free energies of common organic compounds (RH) releasing hydrogen atoms. (**b**) Gibbs free energies of hydroquinones successively releasing two hydrogen atoms in DMSO.

In chemical reactions, HEH_2_ were used to quench the active radicals (R^•^) by donating two hydrogen atoms [35,36], resulting in the formation of stable RH. Given that QH_2_ and QH^•^ are capable of releasing hydrogen atom(s) as antioxidants, they can similarly serve as radical quenchers in radical reactions. Hence, Scheme 5 displays the Gibbs free energies for the release of hydrogen atom(s) from hydroquinones, semiquinones (QH^•^), and common organic compounds (RH) [31,37,38,39] in DMSO to provide a comparative analysis of their thermodynamic feasibility as radical quenchers. By examining these Gibbs free energies from Scheme 5, one can assess and deduce the relative propensity of these compounds to engage in hydrogen atom transfer reactions, which is crucial for their effectiveness in radical quenching roles.

As depicted in Scheme 5, the Gibbs free energies of RH releasing a hydrogen atom range from 76.0 kcal/mol for C_sp3_–H bond in DHA (dihydroanthracene) [31] to 128.1 kcal/mol for C_sp_–H bond in acetylene [37,38,39]. Since the Δ*G*_HR_(QH_2_) and Δ*G*_HR_(QH^•^) values of 118 QH_2_ and QH^•^ range from 63.7 to 87.2 kcal/mol and 50.0 to 81.3 kcal/mol, respectively, this indicates that the majority of QH_2_ and QH^•^ species examined in this work are capable of quenching a broad spectrum of active radicals (R^•^ and 76.0–128.1 kcal/mol) in chemical reactions. Since QH_2_ can release two hydrogen atoms, the Gibbs free energies of two moles R^•^ accepting two moles hydrogen atoms, Δ*G*_2HA_(R^•^), therefore, are estimated to range from 152.0 kcal/mol to 256.2 kcal/mol in DMSO. As discussed and shown in Scheme 5b, the Δ*G*_2HR_(QH_2_) scale of 118 QH_2_ ranges from 114.9 kcal/mol to 167.4 kcal/mol in DMSO, suggesting that 104 QH_2_ with Δ*G*_2HR_(QH_2_) less than 152 kcal/mol are well-suited to quench nearly all the active radicals within the 152.0 to 256.2 kcal/mol range through the two-hydrogen-atoms transfer reaction, QH_2_ + 2R^•^ → Q + 2R−H, driven by a significant negative thermodynamic driving force (Δ*G* << 0 kcal/mol). What is even more interesting is that although certain oxidants such as **115**H_2_ (tetracyano-*o*-benzoquinone, 167.4 kcal/mol) and **40**H_2_ (tetracyano-*p*-benzoquinone, 159.3 kcal/mol) are thermodynamically very weak two-hydrogen-atoms donors, they still possess the potential to act as radical quenchers. They are capable of neutralizing a variety of active radicals, including, but not limited to, PhCH_2_^•^ (87 kcal/mol) [31], O-radicals (174.0–122.7 kcal/mol), Ph^•^ (108.0 kcal/mol) [37,38,39], and HC≡C^•^ (128.1 kcal/mol) [37,38,39], through the release of two hydrogen atoms. These findings reveal the versatility of QH_2_ and QH^•^ as antioxidants and radical quenchers in chemical reactions, highlighting their thermodynamic potential to engage in hydrogen atom transfer (HAT) processes.

### 4.5. Thermodynamic Capabilities of Q and QH^•^ Acting as Hydrogen Atoms Abstractors

**Scheme 6:**
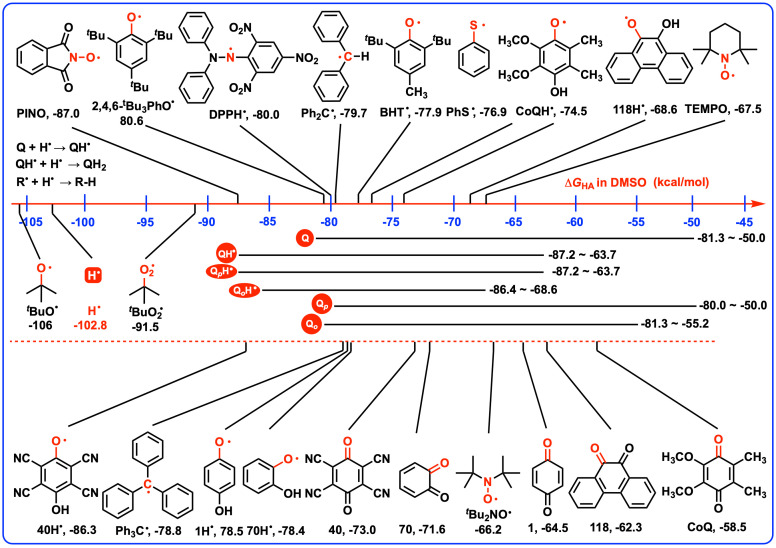
Gibbs free energies of quinones (Q) and semiquinones (QH^•^) accepting hydrogen atoms, as well as common free radicals accepting hydrogen atoms in DMSO.

QH_2_ and QH^•^ can function as antioxidants or radical quenchers by releasing hydrogen atom(s), and conversely, their oxidized forms, Q and QH^•^, can act as hydrogen atoms abstractors to initiate radical reactions. In this context, Scheme 6 compiles the Gibbs free energies of quinones (Q) and semiquinones (QH^•^) accepting hydrogen atoms, as well as common free radicals accepting hydrogen atoms in DMSO.

The capability of certain compounds to act as efficient hydrogen atom abstractors in chemical reactions is characterized by their hydrogen atom affinities (Gibbs free energies of compounds accepting a hydrogen atom). *^t^*BuO^•^ (−106.0 kcal/mol) [31,40,41,42,43] and PINO (phthalimide-*N*-oxyl radical, −87.0 kcal/mol) [31,44,45] are efficient hydrogen atoms abstractors in chemical reactions. If the hydrogen atom affinities of Q or QH^•^, Δ*G*_HA_(Q) or Δ*G*_HA_(QH^•^) are more negative than −85 kcal/mol, the Q or QH^•^ are recognized as thermodynamically strong hydrogens atoms abstractors. Since TEMPO (−67.5 kcal/mol) [31,46,47,48,49] and PhS^•^ (−76.9 kcal/mol) [31,50,51,52] have been reported as medium–strong radical initiators by abstracting active hydrogen atoms, it seems reasonable to define a thermodynamically medium–strong category for hydrogen atom abstractors. Accordingly, if the hydrogen atom affinities of Q and QH^•^ [Δ*G*_HA_(Q) and Δ*G*_HA_(QH^•^)] fall between −65 kcal/mol and −85 kcal/mol, the corresponding Q or QH^•^ are recognized as thermodynamically medium–strong hydrogens atoms abstractors. Finally, if the Δ*G*_HA_(Q) or Δ*G*_HA_(QH^•^) values are more positive than −65 kcal/mol, the Q or QH^•^ are regarded as thermodynamically weak hydrogens atoms abstractors.

This classification system provides a clear framework for evaluating the thermodynamic potential of Q and QH^•^ to initiate radical reactions through hydrogen atom abstraction. Because the Δ*G*_HA_(Q) (−81.3–−50.0 kcal/mol) and Δ*G*_HA_(QH^•^) (−87.2–−63.7 kcal/mol) values are more positive than −87.2 kcal/mol, the thermodynamically hydrogen atoms abstracting capabilities of Q and QH^•^ are considerably weaker compared to efficient abstractors like *^t^*BuO^•^ (−106.0 kcal/mol) [31,40,41,42,43]. For quinones, the Δ*G*_HA_(Q) values range from −81.3 kcal/mol to −50.0 kcal/mol, indicating that 118 quinones fall into the category of thermodynamically weak or medium–strong hydrogen atom abstractors. This suggests a need for careful selection to identify suitable hydrogen atom abstractors. Additionally, DPPH (2,2-diphenyl-1-picrylhydrazyl, −80.0 kcal/mol) [53,54,55,56,57,58] is employed as an important reference material to determine the antioxidant activity of substances by absorbing a hydrogen atom. From a thermodynamic perspective, certain quinones like **41** (−80.0 kcal/mol), **110** (−79.9 kcal/mol), **115** (−81.0 kcal/mol) and **117** (−81.3 kcal/mol) exhibit potential to be used as the alternatives to DPPH in antioxidant determination. Most notably, the Δ*G*_2HA_(Q) values of **41** (−167.2 kcal/mol), **110** (−164.3 kcal/mol), **115** (−167.4 kcal/mol) and **117** (−160.2 kcal/mol) are known in this work; therefore, **41**, **110**, **115** and **117** could also serve as reference materials to determine the BDFE (bond dissociation free energy) of X–H in antioxidant or chemical substances through direct calorimetry.

In contrast to quinones, semiquinone radicals (QH^•^) exhibit an extensive range of thermodynamic capabilities to abstract a hydrogen atom, as indicated by their Δ*G*_HA_(QH^•^) values, which vary from −87.2 kcal/mol to −63.7 kcal/mol. This range encompasses thermodynamically weak, medium–strong, and strong hydrogen atom abstractors. Five semi-9,10-anthraquinone radicals (−63.7–−64.9 kcal/mol), including **59**H^•^, **61**H^•^, **64**H^•^, **65**H^•^ and **66**H^•^, are clarified as the thermodynamically weak hydrogen atom abstractors and may not be suitable for use as such in chemical reactions. On the other end of the spectrum, **40**H^•^ (−86.3 kcal/mol), **41**H^•^ (−87.2 kcal/mol), **115**H^•^ (−86.4 kcal/mol), and **110_b_**H^•^ (−85.5 kcal/mol) are considered thermodynamically strong hydrogen atoms abstractors, which could be used as the alternatives to PINO (−87.0 kcal/mol) [31,44,45] for initiating radical reactions through hydrogen atom abstraction. In summary, the majority of QH^•^ (109 out of 118) are categorized as thermodynamically medium–strong hydrogen atom abstractors, which hold the potential to be used as alternatives to TEMPO (−67.5 kcal/mol) [31,46,47,48,49] or PhS^•^ (−76.9 kcal/mol) [31,50,51,52] for abstracting a hydrogen atom and initiating radical reactions, offering a broad spectrum of applications in the realm of redox chemistry.

## 5. Conclusions

In summary, this work presents a practical thermodynamic dataset that encapsulates the thermodynamic driving forces for 12 distinct chemical processes involving the hydrogenation and dehydrogenation of 118 quinone/hydroquinone couples in DMSO. This valuable dataset is designed for convenient access and practical utility by researchers in the field. Based on the thermodynamic dataset, the thermodynamic capabilities of hydroquinone acting as two-hydrogen-atoms antioxidants and radical quenchers, hydroquinone and semiquinone radicals acting as hydrogen atoms abstractors, and quinone/hydroquinone couples acting as dehydrogenation and hydrogenation reagents, are discussed and explored in detail. The findings and insights are expected to provide a deep understanding of fundamental thermodynamics for quinone/hydroquinone couples in solution, which is instrumental in advancing the extensive application across various fields, including chemical antioxidation and redox reactions.

## Figures and Tables

**Table 1:** Definitions on driving forces of 12 chemical processes for Q/QH_2_ couples accepting or releasing two hydrogen atoms or H_2_ in DMSO.

Hydroquinones Releasing 2H^•^ or H_2_			Quinones Accepting 2H^•^ or H_2_		
*Step X*	Definitions	Parameters	*Step X*	Definitions	Parameters
*Step 1*	QH_2_ → Q_a_H^•^ + H^•^	Δ*G*_HR_(QH_2_)	*Step 7*	Q_a_H^•^ + H^•^ → QH_2_	Δ*G*_HA_(Q_a_H^•^)
*Step 2*	Q_a_H^•^ → Q + H^•^	Δ*G*_HR_(Q_a_H^•^)	*Step 8*	Q + H^•^ → Q_a_H^•^	Δ*G*_HA_(Q)
*Step 3*	QH_2_ → Q_b_H^•^ + H^•^	Δ*G′*_HR_(QH_2_)	*Step 9*	Q_b_H^•^ + H^•^ → QH_2_	Δ*G*_HA_(Q_b_H^•^)
*Step 4*	Q_b_H^•^ → Q + H^•^	Δ*G*_HR_(Q_b_H^•^)	*Step 10*	Q + H^•^ → Q_b_H^•^	Δ*G′*_HA_(Q)
*Step 5*	QH_2_ → Q + 2H^•^	Δ*G*_2HR_(QH_2_)	*Step 11*	Q + 2H^•^ → QH_2_	Δ*G*_2HA_(Q)
*Step 6*	QH_2_ → Q + 2H^•^	Δ*G′*_2HR_(QH_2_)	*Step 12*	Q + 2H^•^ → QH_2_	Δ*G′*_2HA_(Q)

**Table 2:** Expressions of *Equations (1)~(6)* and data sources on fourteen thermodynamic driving forces of quinones and hydroquinones accepting or releasing two hydrogen atoms in DMSO.

Equation X	Expressions	Data Sources
*(1)*	Δ*G*_HR_(QH_2_) = −Δ*G*_HA_(Q_a_H^•^) = Δ*H*_HR_(QH_2_) − 4.9 kcal/mol	Δ*H*_HR_(QH_2_) were calculated by DFT [27]
*(2)*	Δ*G*_HR_(Q_a_H^•^) = −Δ*G*_HA_(Q) = Δ*H*_HR_(Q_a_H^•^) − 4.9 kcal/mol	Δ*H*_HR_(Q_a_H^•^) were calculated by DFT [27]
*(3)*	Δ*G′*_HR_(QH_2_) = −Δ*G*_HA_(Q_b_H^•^) = Δ*H′*_HR_(QH_2_) − 4.9 kcal/mol	Δ*H′*_HR_(QH_2_) were calculated by DFT [27]
*(4)*	Δ*G*_HR_(Q_b_H^•^) = −Δ*G′*_HA_(Q) = Δ*H*_HR_(Q_b_H^•^) − 4.9 kcal/mol	Δ*H*_HR_(Q_b_H^•^) were calculated by DFT [27]
*(5)*	Δ*G*_2HR_(QH_2_) = −Δ*G*_2HA_(Q) = Δ*G*_HR_(QH_2_) + Δ*G*_HR_(Q_a_H^•^)	derived in this work based on Hess’s law
*(6)*	Δ*G′*_2HR_(QH_2_) = −Δ*G′*_2HA_(Q) = Δ*G′*_HR_(QH_2_) + Δ*G*_HR_(Q_b_H^•^)	derived in this work based on Hess’s law

**Table 3:** Thermodynamic driving forces of 118 quinone/hydroquinone couples accepting or releasing two hydrogen atoms on 12 chemical steps in DMSO (unit: kcal/mol).

NO.	R	Δ*G*_HR_(QH_2_) *Step 1*	Δ*G′*_HR_(QH_2_) *Step 3*	Δ*G*_HR_(Q_a_H^•^) *Step 2*	Δ*G*_HR_(Q_b_H^•^) *Step 4*	Δ*G*_2HR_(QH_2_) *Step 5*	Δ*G′*_2HR_(QH_2_) *Step 6*
		−Δ*G*_HA_(Q_a_H^•^) *Step 8*	−Δ*G*_HA_(Q_b_H^•^) *Step 12*	−Δ*G*_HA_(Q) *Step 7*	−Δ*G′*_HA_(Q) *Step 11*	−Δ*G*_2HA_(Q) *Step 9*	−Δ*G′*_2HA_(Q) *Step 10*
1	H	78.5	−	64.5	−	143.0	−
2	N(Me)_2_	70.6	76.7	64.1	57.9	134.7	134.6
3	NH_2_	67.5	74.1	64.8	58.2	132.3	132.3
4	OMe	79.1	76.8	60.1	62.3	139.2	139.1
5	OH	73.2	78.0	65.4	60.6	138.6	138.6
6	SH	74.9	79.2	65.9	61.6	140.8	140.8
7	CH_3_	76.6	77.4	63.2	62.4	139.8	139.8
8	SiH_3_	76.9	79.0	65.8	63.6	142.7	142.6
9	F	78.0	79.5	66.0	64.5	144.0	144.0
10	Cl	79.8	78.2	63.9	65.5	143.7	143.7
11	Br	78.4	79.7	66.3	65.1	144.7	144.8
12	CHO	83.7	85.0	68.8	67.4	152.5	152.4
13	CO_2_Me	82.7	80.8	67.5	69.4	150.2	150.2
14	CF_3_	78.3	81.6	67.6	64.4	145.9	146.0
15	CN	78.8	82.4	69.4	65.9	148.2	148.3
16	OMe	76.9	−	64.4	−	141.3	−
17	CH_3_	75.4	−	61.0	−	136.4	−
18	F	78.8	−	65.1	−	143.9	−
19	Cl	79.1	−	64.6	−	143.7	−
20	CN	83.0	−	69.8	−	152.8	−
21	OMe	73.5	−	60.6	−	134.1	−
22	CH_3_	75.9	−	61.0	−	136.9	−
23	F	78.5	−	64.9	−	143.4	−
24	Cl	79.4	−	64.9	−	144.3	−
25	CN	82.1	−	69.0	−	151.1	−
26	OMe	80.3	79.4	54.1	55.0	134.4	134.4
27	CH_3_	75.1	77.5	61.8	59.4	136.9	136.9
28	F	70.0	73.6	67.1	63.4	137.1	137.0
29	Cl	81.1	77.6	63.1	66.6	144.2	144.2
30	CN	80.4	85.6	72.3	67.1	152.7	152.7
31	OMe	77.3	78.7	57.2	55.8	134.5	134.5
32	CH_3_	76.1	74.7	57.8	59.2	133.9	133.9
33	F	77.8	79.5	65.7	64.0	143.5	143.5
34	Cl	80.2	78.2	63.6	65.6	143.8	143.8
35	CN	83.6	85.5	72.2	70.2	155.8	155.7
36	OMe	74.9	−	60.0	−	134.9	−
37	CH_3_	74.0	−	57.0	−	131.0	−
38	F	78.7	−	65.0	−	143.7	−
39	Cl	79.2	−	64.2	−	143.4	−
40	CN	86.3	−	73.0	−	159.3	−
41	−	87.2	−	80.0	−	167.2	−
42	−	67.6	72.9	67.6	62.2	135.2	135.1
43	−	65.9	−	56.2	−	122.1	−
44	−	67.2	72.3	65.3	60.2	132.5	132.5
45	−	65.1	72.0	66.9	60.0	132.0	132.0
46	−	71.8	−	59.3	−	131.1	−
47	H	72.8	−	57.9	−	130.7	−
48	OMe	69.8	70.4	62.1	61.5	131.9	131.9
49	CH_3_	70.8	72.5	58.9	57.3	129.7	129.8
50	F	72.0	73.3	59.9	58.5	131.9	131.8
51	Cl	72.3	73.9	60.7	59.1	133.0	133.0
52	CN	72.8	77.0	64.4	60.3	137.2	137.3
53	−	70.0	−	58.1	−	128.1	−
54	OMe	70.1	67.6	52.1	54.7	122.2	122.3
55	CH_3_	68.2	69.7	56.9	55.5	125.1	125.2
56	F	69.5	70.2	57.3	56.7	126.8	126.9
57	Cl	69.6	71.4	59.0	57.2	128.6	128.6
58	CN	69.9	74.5	63.1	58.4	133.0	132.9
59	H	64.9	−	50.0	−	114.9	−
60	OMe	69.5	64.6	51.8	56.7	121.3	121.3
61	CH_3_	64.3	64.5	51.7	51.4	116.0	115.9
62	F	66.3	65.1	52.6	53.8	118.9	118.9
63	Cl	66.5	65.2	52.4	53.7	118.9	118.9
64	CN	64.4	65.9	53.5	51.9	117.9	117.8
65	OMe	63.7	63.8	51.3	51.2	115.0	115.0
66	CH_3_	64.3	64.4	51.7	51.6	116.0	116.0
67	F	65.7	65.4	52.3	52.6	118.0	118.0
68	Cl	65.5	65.6	52.8	52.7	118.3	118.3
69	CN	65.5	66.5	54.5	53.5	120.0	120.0
70	H	78.4	−	71.6	−	150.0	−
71	OMe	78.2	81.1	68.4	65.6	146.6	146.7
72	CH_3_	76.9	77.8	71.3	70.3	148.2	148.1
73	F	77.9	81.8	72.8	68.9	150.7	150.7
74	Cl	78.0	79.6	72.5	70.9	150.5	150.5
75	CN	78.0	79.5	73.7	72.3	151.7	151.8
76	OMe	74.9	72.8	66.8	68.9	141.7	141.7
77	CH_3_	77.2	75.9	69.8	71.0	147.0	146.9
78	F	78.0	77.1	70.7	71.5	148.7	148.6
79	Cl	79.0	78.1	71.3	72.2	150.3	150.3
80	CN	81.9	81.8	75.0	75.0	156.9	156.8
81	OMe	78.5	76.9	66.6	68.1	145.1	145.0
82	CH_3_	77.1	76.6	69.6	70.1	146.7	146.7
83	F	77.9	78.4	71.9	71.4	149.8	149.8
84	Cl	78.2	79.0	72.0	71.2	150.2	150.2
85	CN	81.6	82.8	77.5	76.4	159.1	159.2
86	OMe	73.9	79.9	65.1	59.1	139.0	139.0
87	CH_3_	76.1	77.0	69.5	68.6	145.6	145.6
88	F	76.8	79.9	72.9	69.8	149.7	149.7
89	Cl	77.4	80.0	73.2	70.6	150.6	150.6
90	CN	81.3	82.7	76.8	75.4	158.1	158.1
91	OMe	80.1	−	65.5	−	145.6	−
92	CH_3_	75.7	−	70.3	−	146.0	−
93	F	79.1	−	72.5	−	151.6	−
94	Cl	79.1	−	72.0	−	151.1	−
95	CN	80.6	−	74.5	−	155.1	−
96	OMe	77.2	−	55.2	−	132.4	−
97	CH_3_	75.1	−	69.5	−	144.6	−
98	F	77.2	−	70.3	−	147.5	−
99	Cl	78.8	−	72.5	−	151.3	−
100	CN	84.8	−	77.6	−	162.4	−
101	OMe	78.2	74.6	65.7	69.2	143.9	143.8
102	CH_3_	75.2	73.8	68.4	69.9	143.6	143.7
103	F	79.7	78.0	71.2	72.8	150.9	150.8
104	Cl	79.4	78.4	71.3	72.2	150.7	150.6
105	CN	83.9	83.7	77.8	78.0	161.7	161.7
106	OMe	76.0	77.7	60.5	58.8	136.5	136.5
107	CH_3_	73.9	74.4	69.2	68.7	143.1	143.1
108	F	76.8	78.7	71.9	70.0	148.7	148.7
109	Cl	78.2	80.0	73.2	71.4	151.4	151.4
110	CN	84.4	85.5	79.9	78.9	164.3	164.4
111	OMe	73.7	−	64.4	−	138.1	−
112	CH_3_	73.7	−	68.7	−	142.4	−
113	F	78.3	−	71.6	−	149.9	−
114	Cl	79.0	−	72.2	−	151.2	−
115	CN	86.4	−	81.0	−	167.4	−
116	−	71.1	73.1	66.3	64.3	137.4	137.4
117	−	78.9	−	81.3	−	160.2	−
118	−	68.6	−	62.3	−	130.9	−

## Data Availability

The original contributions presented in this study are included in the article/Supplementary Materials.

## Supplementary Materials

The following supporting information can be downloaded at https://www.mdpi.com/article/10.3390/biom15111606/s1, Table S1. Thermodynamic driving forces of 118 hydroquinones releasing two hydrogen atoms in DMSO (unit: kcal/mol); Table S2. The energy values of ΔΔG2HR, ΔΔGHR, ΔΔG′HR, and ΔΔG″HR during the process of hydroquinones releasing two hydrogen atoms.
