# Supplementary material for: Evaluations of Quinone/Hydroquinone Couples Acting as Two Hydrogen Atoms Antioxidants, Radical Quenchers, and Hydrogen Atom Abstractors
Source: Biomolecules. 2025 Nov 15;15(11):1606. doi: 10.3390/biom15111606 (PMC12650150; doi:10.3390/biom15111606)
Supplement: Supplementary file 1 [file biomolecules-15-01606-s001.zip › biomolecules-3952605-supplementary.pdf]

Supplementary Materials

Table S1……………………………………………………………………………………….S2-S5
Table S2……………………………………………………………………………………….S5-S8

Table S1. Thermodynamic driving forces of 118 hydroquinones releasing two hydrogen atoms in DMSO (unit: kcal/mol)

NO.	R	$\Delta H_{HR}(QH_2)$ *Step 1*	$\Delta H'_{HR}(QH_2)$ *Step 3*	$\Delta H_{HR}(Q_aH^{\bullet})$ *Step 2*	$\Delta H_{HR}(Q_bH^{\bullet})$ *Step 4*
1	H	83.4	–	69.4	–
2	$N(Me)_2$	75.5	81.6	69.0	62.8
3	NH_2	72.4	79.0	69.7	63.1
4	OMe	84.0	81.7	65.0	67.2
5	OH	78.1	82.9	70.3	65.5
6	SH	79.8	84.1	70.8	66.5
7	CH_3	81.5	82.3	68.1	67.3
8	SiH_3	81.8	83.9	70.7	68.5
9	F	82.9	84.4	70.9	69.4
10	Cl	84.7	83.1	68.8	70.4
11	Br	83.3	84.6	71.2	70.0
12	CHO	88.6	89.9	73.7	72.3
13	CO_2Me	87.6	85.7	72.4	74.3
14	CF_3	83.2	86.5	72.5	69.3
15	CN	83.7	87.3	74.3	70.8
16	OMe	81.8	–	69.3	–
17	CH_3	80.3	–	65.9	–
18	F	83.7	–	70.0	–
19	Cl	84.0	–	69.5	–
20	CN	87.9	–	74.7	–
21	OMe	78.4	–	65.5	
22	CH_3	80.8	–	65.9	–
23	F	83.4	–	69.8	–
24	Cl	84.3	–	69.8	–
25	CN	87.0	–	73.9	–
26	OMe	85.2	84.3	59.0	59.9
27	CH_3	80.0	82.4	66.7	64.3
28	F	74.9	78.5	72.0	68.3
29	Cl	86.0	82.5	68.0	71.5
30	CN	85.3	90.5	77.2	72.0
31	OMe	82.2	83.6	62.1	60.7
32	CH_3	81.0	79.6	62.7	64.1
33	F	82.7	84.4	70.6	68.9
34	Cl	85.1	83.1	68.5	70.5
35	CN	88.5	90.4	77.1	75.1
36	OMe	79.8	–	64.9	–
37	CH_3	78.9	–	61.9	–

NO.	R	$\Delta H_{HR}(QH_2)$ *Step 1*	$\Delta H'_{HR}(QH_2)$ *Step 3*	$\Delta H_{HR}(Q_aH^{\bullet})$ *Step 2*	$\Delta H_{HR}(Q_bH^{\bullet})$ *Step 4*
38	F	83.6	–	69.9	–
39	Cl	84.1	–	69.1	–
40	CN	91.2	–	77.9	–
41	–	92.1	–	84.9	–
42	–	72.5	77.8	72.5	67.1
43	–	70.8	–	61.1	–
44	–	72.1	77.2	70.2	65.1
45	–	70.0	76.9	71.8	64.9
46	–	76.7	–	64.2	–
47	H	77.7	–	62.8	–
48	OMe	74.7	75.3	67.0	66.4
49	CH_3	75.7	77.4	63.8	62.2
50	F	76.9	78.2	64.8	63.4
51	Cl	77.2	78.8	65.6	64.0
52	CN	77.7	81.9	69.3	65.2
53	–	74.9	–	63.0	–
54	OMe	75.0	72.5	57.0	59.6
55	CH_3	73.1	74.6	61.8	60.4
56	F	74.4	75.1	62.2	61.6
57	Cl	74.5	76.3	63.9	62.1
58	CN	74.8	79.4	68.0	63.3
59	H	69.8	–	54.9	–
60	OMe	74.4	69.5	56.7	61.6
61	CH_3	69.2	69.4	56.6	56.3
62	F	71.2	70.0	57.5	58.7
63	Cl	71.4	70.1	57.3	58.6
64	CN	69.3	70.8	58.4	56.8
65	OMe	68.6	68.7	56.2	56.1
66	CH_3	69.2	69.3	56.6	56.5
67	F	70.6	70.3	57.2	57.5
68	Cl	70.4	70.5	57.7	57.6
69	CN	70.4	71.4	59.4	58.4
70	H	83.3	–	76.5	–
71	OMe	83.1	86.0	73.3	70.5
72	CH_3	81.8	82.7	76.2	75.2
73	F	82.8	86.7	77.7	73.8
74	Cl	82.9	84.5	77.4	75.8
75	CN	82.9	84.4	78.6	77.2
76	OMe	79.8	77.7	71.7	73.8

NO.	R	$\Delta H_{HR}(QH_2)$ *Step 1*	$\Delta H'_{HR}(QH_2)$ *Step 3*	$\Delta H_{HR}(Q_aH^{\bullet})$ *Step 2*	$\Delta H_{HR}(Q_bH^{\bullet})$ *Step 4*
77	CH_3	82.1	80.8	74.7	75.9
78	F	82.9	82.0	75.6	76.4
79	Cl	83.9	83.0	76.2	77.1
80	CN	86.8	86.7	79.9	79.9
81	OMe	83.4	81.8	71.5	73.0
82	CH_3	82.0	81.5	74.5	75.0
83	F	82.8	83.3	76.8	76.3
84	Cl	83.1	83.9	76.9	76.1
85	CN	86.5	87.7	82.4	81.3
86	OMe	78.8	84.8	70.0	64.0
87	CH_3	81.0	81.9	74.4	73.5
88	F	81.7	84.8	77.8	74.7
89	Cl	82.3	84.9	78.1	75.5
90	CN	86.2	87.6	81.7	80.3
91	OMe	85.0	–	70.4	–
92	CH_3	80.6	–	75.2	–
93	F	84.0	–	77.4	–
94	Cl	84.0	–	76.9	–
95	CN	85.5	–	79.4	–
96	OMe	82.1	–	60.1	–
97	CH_3	80.0	–	74.4	–
98	F	82.1	–	75.2	–
99	Cl	83.7	–	77.4	–
100	CN	89.7	–	82.5	–
101	OMe	83.1	79.5	70.6	74.1
102	CH_3	80.1	78.7	73.3	74.8
103	F	84.6	82.9	76.1	77.7
104	Cl	84.3	83.3	76.2	77.1
105	CN	88.8	88.6	82.7	82.9
106	OMe	80.9	82.6	65.4	63.7
107	CH_3	78.8	79.3	74.1	73.6
108	F	81.7	83.6	76.8	74.9
109	Cl	83.1	84.9	78.1	76.3
110	CN	89.3	90.4	84.8	83.8
111	OMe	78.6	–	69.3	–
112	CH_3	78.6	–	73.6	–
113	F	83.2	–	76.5	–
114	Cl	83.9	–	77.1	–
115	CN	91.3	–	85.9	–

NO.	R	$\Delta H_{HR}(QH_2)$ *Step 1*	$\Delta H'_{HR}(QH_2)$ *Step 3*	$\Delta H_{HR}(Q_aH^{\bullet})$ *Step 2*	$\Delta H_{HR}(Q_bH^{\bullet})$ *Step 4*
116	–	76.0	78.0	71.2	69.2
117	–	83.8	–	86.2	–
118	–	73.5	–	67.2	–

Ref. Zhu, X.−Q.; Wang, C.−H.; Liang, H. Scales of Oxidation Potentials, pK_a, and BDE of Various Hydroquinones and Catechols in DMSO. *J. Org. Chem.* **2010**, *75*, 7240 - 7257.

Table S2. The energy values of $\Delta\Delta G_{2HR}$, $\Delta\Delta G_{HR}$, $\Delta\Delta G'_{HR}$, and $\Delta\Delta G''_{HR}$ during the process of hydroquinones releasing two hydrogen atoms

NO.	R	$\Delta\Delta G_{2HR}$	$\Delta\Delta G_{HR}$	$\Delta\Delta G'_{HR}$	$\Delta\Delta G''_{HR}$
1	H	–	−14.0	–	–
2	$N(Me)_2$	0.1	−6.5	−18.8	6.1
3	NH_2	0.0	−2.7	−15.9	6.6
4	OMe	0.1	−19.0	−14.5	−2.3
5	OH	0.0	−7.8	−17.4	4.8
6	SH	0.0	−9.0	−17.6	4.3
7	CH_3	0.0	−13.4	−15.0	0.8
8	SiH_3	0.1	−11.1	−15.4	2.1
9	F	0.0	−12.0	−15.0	1.5
10	Cl	0.0	−15.9	−12.7	−1.6
11	Br	−0.1	−12.1	−14.6	1.3
12	CHO	0.1	−14.9	−17.6	1.3
13	CO_2Me	0.0	−15.2	−11.4	−1.9
14	CF_3	−0.1	−10.7	−17.2	3.3
15	CN	−0.1	−9.4	−16.5	3.6
16	OMe	–	−12.5	–	–
17	CH_3	–	−14.4	–	–
18	F	–	−13.7	–	–
19	Cl	–	−14.5	–	–
20	CN	–	−13.2	–	–
21	OMe	–	−12.9	–	–
22	CH_3	–	−14.9	–	–
23	F	–	−13.6	–	–
24	Cl	–	−14.5	–	–
25	CN	–	−13.1	–	–
26	OMe	0.0	−26.2	−24.4	−0.9
27	CH_3	0.0	−13.3	−18.1	2.4
28	F	0.1	−2.9	−10.2	3.6
29	Cl	0.0	−18.0	−11.0	−3.5
30	CN	0.0	−8.1	−18.5	5.2
31	OMe	0.0	−20.1	−22.9	1.4

NO.	R	$\Delta\Delta G_{2HR}$	$\Delta\Delta G_{HR}$	$\Delta\Delta G'_{HR}$	$\Delta\Delta G''_{HR}$
32	CH_3	0.0	−18.3	−15.5	−1.4
33	F	0.0	−12.1	−15.5	1.7
34	Cl	0.0	−16.6	−12.6	−2.0
35	CN	0.1	−11.4	−15.3	1.9
36	OMe	−	−14.9	−	−
37	CH_3	−	−17.0	−	−
38	F	−	−13.7	−	−
39	Cl	−	−15.0	−	−
40	CN	−	−13.3	−	−
41	−	−	−7.2	−	−
42	−	0.1	0.0	−10.7	5.3
43	−	−	−9.7	−	−
44	−	0.0	−1.9	−12.1	5.1
45	−	0.0	1.8	−12	6.9
46	−	−	−12.5	−	−
47	H	−	−14.9	−	−
48	OMe	0.0	−7.7	−8.9	0.6
49	CH_3	−0.1	−11.9	−15.2	1.7
50	F	0.1	−12.1	−14.8	1.3
51	Cl	0.0	−11.6	−14.8	1.6
52	CN	−0.1	−8.4	−16.7	4.2
53	−	−	−11.9	−	−
54	OMe	−0.1	−18.0	−12.9	−2.5
55	CH_3	−0.1	−11.3	−14.2	1.5
56	F	−0.1	−12.2	−13.5	0.7
57	Cl	0.0	−10.6	−14.2	1.8
58	CN	0.1	−6.8	−16.1	4.6
59	H	−	−14.9	−	−
60	OMe	0.0	−17.7	−7.9	−4.9
61	CH_3	0.1	−12.6	−13.1	0.2
62	F	0.0	−13.7	−11.3	−1.2
63	Cl	0.0	−14.1	−11.5	−1.3
64	CN	0.1	−10.9	−14.0	1.5
65	OMe	0.0	−12.4	−12.6	0.1
66	CH_3	0.0	−12.6	−12.8	0.1
67	F	0.0	−13.4	−12.8	−0.3
68	Cl	0.0	−12.7	−12.9	0.1
69	CN	0.0	−11.0	−13.0	1.0
70	H	−	−6.8	−	−

NO.	R	$\Delta\Delta G_{2HR}$	$\Delta\Delta G_{HR}$	$\Delta\Delta G'_{HR}$	$\Delta\Delta G''_{HR}$
71	OMe	−0.1	−9.8	−15.5	2.9
72	CH_3	0.1	−5.6	−7.5	0.9
73	F	0.0	−5.1	−12.9	3.9
74	Cl	0.0	−5.5	−8.7	1.6
75	CN	−0.1	−4.3	−7.2	1.5
76	OMe	0.0	−8.1	−3.9	−2.1
77	CH_3	0.1	−7.4	−4.9	−1.3
78	F	0.1	−7.3	−5.6	−0.9
79	Cl	0.0	−7.7	−5.9	−0.9
80	CN	0.1	−6.9	−6.8	−0.1
81	OMe	0.1	−11.9	−8.8	−1.6
82	CH_3	0.0	−7.5	−6.5	−0.5
83	F	0.0	−6.0	−7.0	0.5
84	Cl	0.0	−6.2	−7.8	0.8
85	CN	−0.1	−4.1	−6.4	1.2
86	OMe	0.0	−8.8	−20.8	6.0
87	CH_3	0.0	−6.6	−8.4	0.9
88	F	0.0	−3.9	−10.1	3.1
89	Cl	0.0	−4.2	−9.4	2.6
90	CN	0.0	−4.5	−7.3	1.4
91	OMe	−	−14.6	−	−
92	CH_3	−	−5.4	−	−
93	F	−	−6.6	−	−
94	Cl	−	−7.1	−	−
95	CN	−	−6.1	−	−
96	OMe	−	−22.0	−	−
97	CH_3	−	−5.6	−	−
98	F	−	−6.9	−	−
99	Cl	−	−6.3	−	−
100	CN	−	−7.2	−	−
101	OMe	0.1	−12.5	−5.4	−3.6
102	CH_3	−0.1	−6.8	−3.9	−1.4
103	F	0.1	−8.5	−5.2	−1.7
104	Cl	0.1	−8.1	−6.2	−1.0
105	CN	0.0	−6.1	−5.7	−0.2
106	OMe	0.0	−15.5	−18.9	1.7
107	CH_3	0.0	−4.7	−5.7	0.5
108	F	0.0	−4.9	−8.7	1.9
109	Cl	0.0	−5.0	−8.6	1.8

NO.	R	$\Delta\Delta G_{2HR}$	$\Delta\Delta G_{HR}$	$\Delta\Delta G'_{HR}$	$\Delta\Delta G''_{HR}$
110	CN	−0.1	−4.5	−6.6	1.1
111	OMe	−	−9.3	−	−
112	CH_3	−	−5.0	−	−
113	F	−	−6.7	−	−
114	Cl	−	−6.8	−	−
115	CN	−	−5.4	−	−
116	−	0.0	−4.8	−8.8	2.0
117	−	−	2.4	−	−
118	−	−	−6.3	−	−

$\Delta\Delta G_{2H} = \Delta G'_{2HR}(QH_2) - \Delta G_{2HR}(QH_2)$, $\Delta\Delta G_{HR} = \Delta G_{HR}(Q_aH^\bullet) - \Delta G_{HR}(QH_2)$, $\Delta\Delta G'_{HR} = \Delta G_{HR}(Q_bH^\bullet) - \Delta G'_{HR}(QH_2)$, and $\Delta\Delta G''_{HR} = \Delta G'_{HR}(QH_2) - \Delta G_{HR}(QH_2)$.
